# Willingness to Use and Pay for Digital Health Care Services According to 4 Scenarios: Results from a National Survey

**DOI:** 10.2196/40834

**Published:** 2023-03-29

**Authors:** Junbok Lee, Yumi Oh, Meelim Kim, Belong Cho, Jaeyong Shin

**Affiliations:** 1 Health-IT Center Yonsei University Health System Seoul Republic of Korea; 2 Department of Human Systems Medicine Seoul National University College of Medicine Seoul Republic of Korea; 3 Korea Health Promotion Institute Seoul Republic of Korea; 4 Herbert Wertheim School of Public Health and Human Longevity Science University of California San Diego San Diego, CA United States; 5 Department of Family Medicine Seoul National University Hospital Seoul Republic of Korea; 6 Department of Preventive Medicine Yonsei University College of Medicine Seoul Republic of Korea; 7 Institute of Health Services Research Yonsei University Seoul Republic of Korea; 8 Institute for Innovation in Digital Healthcare Yonsei University Seoul Republic of Korea

**Keywords:** digital health intervention, service experience, willingness to pay, willingness to use, digital health, health technology

## Abstract

**Background:**

Smartphones and their associated technology have evolved to an extent where these devices can be used to provide digital health interventions. However, few studies have been conducted on the willingness to use (WTU) and willingness to pay (WTP) for digital health interventions.

**Objective:**

The purpose of this study was to investigate how previous service experience, the content of the services, and individuals’ health status affect WTU and WTP.

**Methods:**

We conducted a nationwide web-based survey in 3 groups: nonusers (n=506), public service users (n=368), and private service users (n=266). Participants read scenarios about an imagined health status (such as having a chronic illness) and the use of digital health intervention models (self-management, expert management, and medical management). They were then asked to respond to questions on WTU and WTP.

**Results:**

Public service users had a greater intention to use digital health intervention services than nonusers and private service users: scenario A (health-risk situation and self-management), nonusers=odd ratio [OR] .239 (SE .076; *P*<.001) and private service users=OR .138 (SE .044; *P*<.001); scenario B (health-risk situation and expert management), nonusers=OR .175 (SE .040; *P*<.001) and private service users=OR .219 (SE .053; *P*<.001); scenario C (chronic disease situation and expert management), nonusers=OR .413 (SE .094; *P*<.001) and private service users=OR .401 (SE .098; *P*<.001); and scenario D (chronic disease situation and medical management), nonusers=OR .480 (SE .120; *P*=.003) and private service users=OR .345 (SE .089; *P*<.001). In terms of WTP, in scenarios A and B, those who used the public and private services had a higher WTP than those who did not (scenario A: β=–.397, SE .091; *P*<.001; scenario B: β=–.486, SE .098; *P*<.001). In scenario C, private service users had greater WTP than public service users (β=.264, SE .114; *P*=.02), whereas public service users had greater WTP than nonusers (β=–.336, SE .096; *P*<.001). In scenario D, private service users were more WTP for the service than nonusers (β=–.286, SE .092; *P*=.002).

**Conclusions:**

We confirmed that the WTU and WTP for digital health interventions differed based on individuals’ prior experience with health care services, health status, and demographics. Recently, many discussions have been made to expand digital health care beyond the early adapters and fully into people’s daily lives. Thus, more understanding of people’s awareness and acceptance of digital health care is needed.

## Introduction

Since their introduction, smartphones and their associated technology have evolved to an extent where these devices can be used to provide personal health care services through various mobile apps [1-4]. The number of such apps continues to increase [5,6].

Through such digital health interventions, people can manage their health anytime and anywhere [7,8]. Additionally, digital health interventions have the advantage of enabling health management through features that use objective, numerical, and health-related data, such as the step counter and heart rate tracker [9]. Digital health interventions aid in continuous health management, strengthening the potential to prevent chronic diseases by promoting constant individual health monitoring and to reduce medical expenses [10,11]. Interest in these interventions has further increased because of the COVID-19 pandemic and the consequent restrictions on outdoor activities and movements [12-15]. Given the growing preference for services that do not require physical contact, digital health interventions will only become more prevalent [16]. Digital health interventions are being developed for various medical conditions to complement traditional medical care and improve patient experience [17,18]. The US Food and Drug Administration approved several digital health apps, such as those for the management of diabetes (BlueStar) or the treatment of substance use disorder (reSET) [19]. In Germany, digital health apps approved by BfArM (*Bundesamt für Arzneimittel und Medizinprodukte*; the German Federal Institute for Drugs and Medical Devices) could be included in the DiGA (*digitale Gesundheitsanwendung*; digital health applications) directory for reimbursement [20].

Even with the convenience, usefulness, and potential for future development of digital health interventions, only some people manage their health using digital health care tools [21,22]. Additionally, the retention rate of digital health intervention services is low [23-25]. It is necessary to provide opportunities for more people to experience the service and make them use the service continuously. Thus, it is vital to identify factors that affect people’s willingness to use (WTU) and willingness to pay (WTP) for these services.

Only a few studies have been conducted on the WTU and WTP for digital health interventions [26-30]. Previous studies have identified demographic and health-related factors that affect the WTP for digital health interventions [26]. Research showed that the absolute WTP of those in the UK-representative cohort was £196 (US $258) and the marginal WTP was £160 (US $211), whereas those who availed the national digital health program had an absolute WTP of £162 (US $214) and a marginal WTP of £151 (US $199). Another study conducted an experimental vignette to identify factors affecting people’s use of and payment for mobile health care apps in the context of 4 different business models [27]. It showed that doctors’ recommendations helped increase both the WTU and WTP in Germany and the Netherlands.

This study intended to investigate how individuals’ previous service experience, the content of the services, and health status influence the WTU and WTP of digital health interventions. Referring to previous research [26], we surveyed not only those who availed public digital health intervention services but also those who had experience with private services in South Korea. We subdivided digital health interventions into self-management, expert (nonmedical) management, and medical personnel management to identify differences by service type.

## Methods

### Digital Health Interventions: Public and Private Service

Mobile Healthcare at public health centers is a free health care service program provided by the South Korean government. The service team at public health centers helps individuals manage their daily lives by setting health goals and counseling them via smartphones with activity trackers. As part of the program, they visit the public health centers for counseling and examinations and revisit after 3 and 6 months for check-ups.

Company N’s digital health intervention is a mobile-based app service whose users aim to lose weight and prevent diabetes through lifestyle changes. Based on behavioral science and psychology, health care coaches communicate with users to set health care goals and provide nutrition and exercise feedback, which help them achieve those goals. This service is used in the Centers for Disease Control and Prevention’s Diabetes Prevention Program in the United States.

### Participants

For this study, a nationwide web-based and mobile survey of people aged 19 to 59 years was conducted. We recruited participants from 3 groups: nonusers (n=506), public service users (n=368), and private service users (n=266). Public service users were participants who took part in the Mobile Healthcare program at public health centers. Private service users were people who experienced Company N’s digital health intervention. In the case of public and private service users, recruitment notices were posted on the notice board of the mobile apps. People who expressed their intention to participate in the survey received a survey link. Nonusers were recruited via emails to a large-scale web-based panel of a research company. Based on the Mobile Healthcare project promoted by the Korean Ministry of Health and Welfare, samples of public service users were recruited using a proportional allocation of gender, age, and residence in 2020. Nonservice users were sampled using proportional rates based on gender, age, and residence as of 2020 in South Korea for national representation. All participants responded through a web page developed by the research company, and the data were stored in the research company’s database. The participants received ₩1500 (South Korean won; US $1=₩1100) as a reward for the survey. Table 1 shows the demographic distribution of the study participants.

**Table 1:** Demographic distribution of the participants.

				Nonuser (n=506)		Public service user (n=368)		Private service user (n=266)
**Demographics**								
	**Age range (years), n (%)**							
		19-29	121 (23.9)		23 (6.3)		64 (24.1)	
		30-39	113 (22.3)		92 (25)		78 (29.3)	
		40-49	136 (26.9)		160 (43.5)		72 (27.1)	
		≥50	136 (26.9)		93 (25.3)		52 (19.5)	
	**Gender, n (%)**							
		Men	258 (51)		112 (30.4)		122 (45.9)	
		Women	248 (49)		256 (69.6)		144 (54.1)	
	**Residence, n (%)**							
		Seoul Capital Area	268 (53)		76 (20.7)		80 (30.1)	
		Others	238 (47)		292 (79.3)		186 (69.9)	
**Health status**								
	**Medication, n (%)**							
		Yes	160 (31.6)		45 (12.2)		57 (21.4)	
		No	346 (68.4)		323 (87.8)		209 (78.6)	
	**Hypertension or diabetes, n (%)**							
		Yes	119 (23.5)		56 (15.2)		41 (15.4)	
		No	387 (76.5)		312 (84.8)		225 (84.6)	

### Design and Procedure

People’s WTU and WTP may change according to the contents of digital health interventions, and several factors can influence them. Therefore, in this study, we created 3 digital health intervention models referring to the Evidence Standards Framework developed by The National Institute for Health and Care Excellence in the United Kingdom [31]. According to the functional aspect and potential risks, this framework classifies the level of digital health technology (DHT) into tier 1, tier 2, tier 3a, and tier 3b. Tier 1 includes DHT that provides systemic benefits but no direct benefits to the patient. Tier 2 includes DHT that cannot evaluate a patient’s health outcomes but can help them live a healthy life by providing information and offering simple monitoring services based on the patient’s health-related data. Tier 3a is a service for preventive behavioral modifications and management (which allows users to record and selectively exchange data with specialists) designed to modify health behaviors and eating habits using DHT. Tier 3b refers to DHT with measurable improvements, such as treatment and diagnosis devices. These include devices that provide treatment for diagnosed diseases, provide automated information records and data to experts, and perform calculations that affect clinical decisions. Tier 1 was excluded from this study because it does not directly serve patients.

All the study participants read the scenarios (Textbox 1) and responded to the questions (Table 2). They first read situation scenarios about their health status to imagine themselves as part of a high-risk group. Subsequently, they read the content of digital health interventions for self-management and then indicated their WTU and WTP. Thereafter, they read the scenario for digital health interventions administered by a health care professional (nonmedical person) and responded with their WTU and WTP in the same way.

Next, the participants read the chronic disease patient scenario to imagine themselves as patients with a chronic disease. They read the content of digital health interventions offered by a health care professional (nonmedical person; same as the previous service scenario) and responded with their WTU and WTP. Lastly, they responded with their WTU and WTP for the mobile app that verified the treatment effect and was managed by a doctor.

**Text of the scenarios.**

**Health-risk situation**

- Imagine that a routine medical check-up reveals that you are at a high risk of becoming diabetic. The doctor advises you to come back for a check-up after three months of regular exercising and eating a healthy diet instead of prescribing medication.

**Self-management**

- A service that allows people to enter and monitor health-related data weekly or monthly, such as their food intake, steps walked, weight, blood pressure level, pulse rate, blood sugar level, and so on.

**Expert management**

- A service wherein a healthcare expert (non-medical person) sets up an exercise and diet plan based on the health-related information (diet, weight, etc.) provided, and sends messages via the application on a regular basis for counselling, or to share educational information and advice.

**Chronic disease situation**

- Imagine that you started taking diabetes medicine because your fasting blood sugar level did not drop, and the doctor recommended also utilizing the suggested service.

**Expert management**

- A service wherein a healthcare expert (non-medical person) sets up an exercise and diet plan based on the health-related information (diet, weight…) provided, and sends messages via the application on a regular basis for counselling, or to share educational information and advice.

**Medical management**

- A mobile application-based service that proves the effectiveness of diabetes treatment and a doctor checks the medical data entered by the patient undergoing treatment as well as provides a customized exercise and diet plan.

**Table 2:** Scenario design.

	Self -management (tier 2)	Expert management (tier 3a)	Medical management (tier 3b)
Health-risk situation	Scenario A	Scenario B	N/A^a^
Chronic disease situation	N/A	Scenario C	Scenario D

^a^N/A: not applicable.

### Covariates

#### Sociodemographic and Health Status

Before reading the scenarios, the participants provided information about their age, gender, and residence. After the survey ended, they provided information about their income and occupation. They also indicated their subjective health status, diagnosed disease (high blood pressure, diabetes, etc), and whether they had taken medication for 3 months or longer within the last year for their disease.

#### Dependent Variables, WTU, and WTP Questions

The participants read 4 scenarios (A to D) and indicated their WTU the health care service app in each scenario on a 4-point scale (1=not at all, 2=not very much, 3=somewhat willing, and 4=highly willing). Participants who responded with “somewhat” and “a lot” were asked how much they were WTP per month for the service.

### Statistical Analysis

In this study, logistic regression was used to identify factors affecting the WTU digital health interventions with completed questionnaires. Multiple linear regression analysis was conducted to confirm the WTP. Regarding the WTP, the analysis was performed by log transformation. We also conducted an ANOVA to find out the difference between participants’ WTU and WTP according to their service experience. STATA (version 16; StataCorp) software was used for the analysis.

### Ethics Approval

This study was approved by the institutional review board of the Korea Health Promotion Institute (120160811107AN01-2020-HR-049-02). All participants agreed to participate in the study after reading the explanation page, including the purpose of this study, the number of participants, and the data storage period.

## Results

### WTP

In scenario A, the average WTP was ₩21,909 (SD ₩22,418) for private service users, ₩17,020 (SD ₩15,877) for public service users, and ₩11,913 (SD ₩11,090) for nonusers. In scenario B, the average WTP was ₩17,636 (SD ₩15,356) for private service users, ₩15,392 (SD ₩15,278) for public service users, and ₩10,279 (SD ₩10,428) for nonusers. In scenario C, the average WTP was ₩21,322 (SD ₩21,836) for private service users, ₩14,516 (SD ₩14,977) for public service users, and ₩11,906 (SD ₩12,268) for nonusers. In scenario D, the average WTP was ₩23,520 (SD ₩25,277) for private service users, ₩15,500 (SD ₩16,035) for public service users, and ₩13,084 (SD ₩13,288) for nonusers. Table 3 shows the summary statistics of the WTP.

**Table 3:** Willingness to pay (₩; US $1=₩1100).

		Nonuser (n=506), ₩ (US $)	Public service user (n=368), ₩ (US $)	Private service user (n=266), ₩ (US $)
**Scenario A**				
	Mean	11,913 (10.8)	17,020 (15.5)	21,909 (19.9)
	SD	11,090 (10.1)	15,877 (14.4)	22,418 (20.4)
	Median	10,000 (9.1)	10,000 (9.1)	14,000 (12.7)
	Range	0-65,000 (0-59.1)	0-100,000 (0-90.9)	0-109,000 (0-99.1)
**Scenario B**				
	Mean	10,279 (9.3)	15,392 (14.0)	17,636 (16.0)
	SD	10,428 (9.5)	15,278 (13.9)	15,356 (14.0)
	Median	7000 (6.4)	10,000 (9.1)	10,000 (9.1)
	Range	0-55,000 (0-50)	0-90,000 (0-81.8)	0-80,000 (72.7)
**Scenario C**				
	Mean	11,906 (10.8)	14,516 (13.2)	21,322 (19.4)
	SD	12,268 (11.2)	14,977 (13.6)	21,836 (19.9)
	Median	8000 (7.3)	10,000 (9.1)	11,000 (10.0)
	Range	0-70,000 (0-63.6)	0-90,000 (0-81.8)	0-100,000 (0-90.9)
**Scenario D**				
	Mean	13,084 (11.9)	15,500 (14.1)	23,520 (21.4)
	SD	13,288 (12.1)	16,035 (14.6)	25,277 (23.0)
	Median	10,000 (9.1)	10,000 (9.1)	15,000 (13.6)
	Range	0-80,000 (0-72.7)	0-100,000 (0-90.9)	0-150,000 (0-136.4)

### WTU and WTP in Scenario A (Health-Risk Situation and Self-management)

We explored the WTU and WTP of those in the health-risk situation on the self-management service (see Multimedia Appendix 1). Linear regression analyses were conducted to identify the factors influencing the WTU digital health interventions. In scenario A, age, gender, income, type of service experience, medication use, and residence affected the WTU. Specifically, younger people (odds ratio [OR] .961, SE .010; *P*<.001), women (OR .470, SE .099; *P*<.001), people with high income (OR 1.364, SE .099; *P*<.001), and people who lived in the metropolitan area (OR .629, SE .144; *P*=.04) were more WTU the self-management service. In the case of service experience, public service users had a greater intention to use the self-management service than nonusers and private service users (nonusers: OR .239, SE .076; *P*<.001; private service users: OR .138, SE .044; *P*<.001). People who were on medication for 1 year were more likely to use the self-management service (OR 3.171, SE 1.082; *P*=.001). The explanatory power of the model with all predictors was 13.1%. We also conducted logistic regression analyses to examine the WTP in scenario A. Those who used the public and private services had a higher WTP than those who did not (β=–.397, SE .091; *P*<.001). The explanatory power of WTP was 5.2%.

### WTU and WTP in Scenario B (Health-Risk Situation and Expert Management)

We investigated the WTU and WTP of those in the health-risk situation on the expert management service (see Multimedia Appendix 2). The type of service experience and medication use affected the WTU in scenario B. Specifically, public service users showed greater intention to use the service than private service users and nonusers (nonusers: OR .175, SE .040; *P*<.001; private service users: OR .219, SE .053; *P*<.001). People who were on medication for 1 year were more WTU the expert management service (OR 1.773, SE .365; *P*=.005). The explanatory power of the model with all predictors was 7.2%. We also performed logistic regression analyses to examine the WTP. Private and public service users had higher WTP than nonusers (β=–.486, SE .098; *P*<.001). In terms of demographics, women had a greater WTP for the service than men (β=.233, SE .082; *P*=.005). The explanatory power of WTP was 5.9%.

### WTU and WTP in Scenario C (Chronic Patient Situation and Expert Management)

We conducted linear regression analyses to explore the WTU in scenario C (see Multimedia Appendix 3). Several factors influenced the WTU: age, gender, type of service experience, and having high blood pressure or diabetes. To be specific, younger people (β=.982, SE .008; *P*=.03) and women (β=.705, SE .119; *P*=.04) were more WTU the service. In the case of service experience, public service users showed greater WTU the expert management service than nonusers and private service users (nonusers: OR .413, SE .094; *P*<.001; private service users: OR .401, SE .098; *P*<.001). Regarding health status, those with high blood pressure or diabetes showed more WTU the service (OR 1.751, SE .487; *P*=.04). The explanatory power of the model with all predictors was 3.4%. The result of logistic analyses of the WTP showed that private service users had greater intention to pay than public service users (β=.264, SE .114; *P*=.02), whereas public service users had greater WTP than nonusers (β=–.336, SE .096; *P*<.001). Women were more WTU for the service than men (β=.250, SE .080; *P*=.002), similar to scenario B. The explanatory power of the WTP was 5.4%.

### WTU and WTP in Scenario D (Chronic Patient Situation and Medical Management)

We performed linear regression analyses to investigate the WTU of those in the chronic disease situation on the medical management service (see Multimedia Appendix 4). Age, gender, type of service experience, and having high blood pressure or diabetes influenced the WTU in scenario D. Specifically, younger people (OR .967, SE .009; *P*<.001) and women (OR .569, SE .106; *P*=.002) were more WTU the service. Similar to the other scenarios, public service users showed greater WTU the medical management service (nonusers: OR .480, SE .120; *P*=.003; private service users: OR .345, SE .089; *P*<.001). Those with high blood pressure or diabetes had higher intention to use the service (OR 1.894, SE .596; *P*=.04). The explanatory power of the model with all predictors was 5.2%. The result of logistic analyses of the WTP revealed that private and public service users were more WTP for the service than nonusers (β=–.286, SE .092; *P*=.002). Those who experienced private services had a marginally higher WTP than those who used public services (β=.193, SE .111; *P*=.08). Younger people (β=–.010, SE .004; *P*=.007) and women (β=.177, SE .779; *P*=.02) had a higher likelihood of paying for the medical management service. The explanatory power of the model for WTP was 4.4%.

## Discussion

### Principal Findings

We conducted a web-based survey to investigate the WTU and WTP according to the type of digital health intervention, wherein the respondents were divided into 3 groups (nonusers, public service users, and private service users). We also aimed to identify the factors that affect the WTU and WTP for digital health interventions.

Participants’ WTU and WTP for digital health interventions differed significantly based on their prior experience with health care services. Public service users tended to use digital health intervention more than nonusers and private service users, whereas private service users were more WTP for digital health interventions than the others. This trend was true for all 4 scenarios. Private service users had an average WTP that is 1.5 to 2 times higher than nonusers. However, in this study, it is not clear whether those with high WTP used private services or whether their WTP increased because of positive experiences with the services. Public service users were 1.2 to 1.5 times more WTP than nonusers. In the health-risk situation, there was no difference in the WTP between public and private service users, but the WTP of private service users was much higher than that of public service users in the chronic disease situation.

At first, we expected that private service users would have a higher intention to use digital health interventions. Contrary to our expectations, public services users showed higher WTU such interventions. This might imply higher motivation and interest in terms of health care among public service users. They must have visited the public health center at least thrice to avail the service for additional examination and counseling and receive activity trackers. As the public service is linked to the national health examination, perhaps they realized how severe their health condition was and felt the need for health care. Hence, they showed high intention to use digital health interventions in our study.

An individual’s health status is one of the most critical aspects of the WTP for digital health interventions. Even when the same service was to be provided by a nonmedical person (scenarios B and C), participants were WTP more after reading scenario C (chronic disease situation) than in scenario B (health-risk situation). Additionally, in the health-risk situation scenarios (A and B), having high blood pressure and diabetes did not affect their WTU digital health interventions. Rather, participants with such ailments were more WTU digital health interventions than the others in the chronic disease situation scenarios (C and D).

The content of the services provided is a factor that affects people’s WTP and WTU. This scenario was developed based on The National Institute for Health and Care Excellence’s Evidence Standards Framework. Scenario A is a self-care service that allows people to manage their health. Services in scenarios B and C help people manage exercise and diet with health care experts. In contrast, scenario D is a service in which medical professionals manage diseases with services verified to be effective. Participants wanted to use and pay more for the service in scenario D than in scenarios B and C. This result shows that the more professional and advanced the service, the more willing people are to use and pay for the service.

In scenario D, the importance of validating effectiveness in digital health care services was confirmed. Compared to other scenarios, people were WTU and WTP more for the service that validated their effectiveness. The previous study showed that the effectiveness of digital health care services is an essential factor for British health professionals [32]. Our result also indicated that service users also recognized the importance of effectiveness by clinical evidence.

Another factor highlighted in this study is the percentage of people who are WTP for services. Similar previous studies, the proportion of people WTP for services was at the level of 50% to 60%, and the rest were unwilling to pay [26]. This result indicates that it is necessary to work on the maturation of DHT.

Like previous research [26,27,29], this study showed that age, gender, and income affected the WTP for digital health interventions. Younger individuals and men were more WTP for digital health intervention services. However, the results regarding the WTU somewhat differed from previous studies. Women were more WTU digital health interventions than men in scenarios A, C, and D.

### Limitation

First, the participants responded to hypothetical scenarios, which means that they answered based on what they imagined about a given situation. Their response to a similar real-life situation may distinctly differ. Their reactions to similar situations in practice may be different because of the service’s various features that determine the price, such as governmental regulatory approval with significant evidence or the existence of a physician guide. Additionally, this study cited diabetes as an example of a chronic disease. Patients with other serious diseases may want to pay more for services. Despite this limitation, this study might help provide a lot of insight into developing user-centered services. Second, different countries have different health insurance systems, so the WTU and WTP in other nations may differ from the result of this study. The WTP presented in this study is the general price average for hypothetical scenarios, and attention is needed to interpret it directly. Third, the WTP in scenario A was higher than those in other scenarios. This may be attributed to the question order bias. To reduce this bias, the order of scenarios A to D could have been presented at random. However, we did not do so because we had to consider the presence or absence of disease and services. Fourth, the explanatory power of the regression analysis was not high. However, even in a previous study [27], the explanatory power of the WTP was 3% to 8%, close to the WTP explanatory power of 4.4% to 5.9% for scenarios A to D in this study.

### Conclusion

Digital health care technology has continued to develop and is expected to grow further. More people are WTU their smartphones to manage their health, creating various health care innovations. Recently, there have been many discussions about expanding digital health care for general people, not only early adopters. However, studies have yet to be conducted on the WTU and WTP for digital health care. It is necessary to develop a deeper understanding of people’s awareness and acceptance of digital health care. Digital health care companies should develop their product based on this understanding. Since digital health care needs to work within the health care system, it is essential to evaluate the effectiveness of the services with clinical evidence.

## Appendix

Multimedia Appendix 1 Willingness to use (WTU) and willingness to pay (WTP) in scenario A (health-risk situation and self-management).

Multimedia Appendix 2 Willingness to use (WTU) and willingness to pay (WTP) in scenario B (health-risk situation and expert management).

Multimedia Appendix 3 Willingness to use (WTU) and willingness to pay (WTP) in scenario C (chronic disease situation and expert management).

Multimedia Appendix 4 Willingness to use (WTU) and willingness to pay (WTP) in scenario D (chronic disease situation and medical management).

## Abbreviations

BfArM: Bundesamt für Arzneimittel und Medizinprodukte (German Federal Institute for Drugs and Medical Devices)
DiGA: digitale Gesundheitsanwendung (digital health applications)
DHT: digital health technology
OR: odds ratio
WTP: willingness to pay
WTU: willingness to use
