# Supplementary material for: Willingness to Use and Pay for Digital Health Care Services According to 4 Scenarios: Results from a National Survey
Source: JMIR Mhealth Uhealth. 2023 Mar 29;11:e40834. doi: 10.2196/40834 (PMC10131682; doi:10.2196/40834)
Supplement: Multimedia Appendix 1 [file mhealth_v11i1e40834_app1.docx]

**Multimedia Appendix 1**

WTU and WTP on Scenario A (Health risk situation & Self-management)

|  | **Willing to Use** | | | |  | **Willing to Pay** | | | |
| --- | --- | --- | --- | --- | --- | --- | --- | --- | --- |
|  | **OR(SE)** | **z** | **P** | **95% CI** |  | **Coef.(SE)** | **t** | **P** | **95% CI** |
| **Demographics** |  |  |  |  |  |  |  |  |  |
| **Age** | .961 (.010) | -3.77 | .000 | .941 to .981 |  | -.003 (.004) | -.78 | .438 | -.010 to .005 |
| **Gender** | .470 (.099) | -3.57 | .000 | .311 to .712 |  | .120 (.076) | 1.59 | .112 | -.028 to .269 |
| **Income** | 1.364 (.099) | 4.28 | .000 | 1.183 to 1.572 |  | .022 (.025) | .89 | .375 | -.027 to .071 |
| **Residence** | .629 (.144) | -2.03 | .043 | .042 to .985 |  | .027 (.078) | .57 | .726 | -.125 to .179 |
| **Service Experience** |  |  |  |  |  |  |  |  |  |
| **Non-User** | .239 (.076) | -4.52 | .000 | .129 to .445 |  | -.397 (.091) | -4.36 | .000 | -.576 to -.219 |
| **Private Service User** | .138 (.044) | -6.16 | .000 | .074 to .259 |  | .159 (.110) | 1.45 | .146 | -.059 to .375 |
| **Health Status** |  |  |  |  |  |  |  |  |  |
| **Medication** | 3.171 (1.082) | 3.38 | .001 | 1.625 to 6.190 |  | .031 (.090) | .34 | .733 | -.147 to .209 |
| **High Blood Pressure, Diabetes** | 1.434 (.511) | 1.01 | .312 | .713 to 2.884 |  | .059 (.104) | .57 | .570 | -.145 to .264 |
