# Supplementary material for: Willingness to Use and Pay for Digital Health Care Services According to 4 Scenarios: Results from a National Survey
Source: JMIR Mhealth Uhealth. 2023 Mar 29;11:e40834. doi: 10.2196/40834 (PMC10131682; doi:10.2196/40834)
Supplement: Multimedia Appendix 2 [file mhealth_v11i1e40834_app2.docx]

**Multimedia Appendix 2**

WTU and WTP on Scenario B (Health risk situation & Expert management)

|  | **Willing to Use** | | | |  | **Willing to Pay** | | | |
| --- | --- | --- | --- | --- | --- | --- | --- | --- | --- |
|  | **OR(SE)** | **z** | **P** | **95% CI** |  | **Coef.(SE)** | **t** | **P** | **95% CI** |
| **Demographics** |  |  |  |  |  |  |  |  |  |
| **Age** | .992 (.007) | -1.01 | .310 | .978 to 1.007 |  | -.003 (.004) | -.61 | .539 | -.018 to .006 |
| **Gender** | .808 (.123) | -1.40 | .161 | .599 to 1.089 |  | .233 (.082) | 2.84 | .005 | -.072 to .395 |
| **Income** | 1.065 (.054) | 1.24 | .214 | .964 to 1.175 |  | -.002 (.027) | -.06 | .955 | -.054 to .051 |
| **Residence** | .773 (.124) | -1.60 | .109 | .564 to 1.059 |  | -.074 (.083) | -.88 | .378 | -.237 to .090 |
| **Service Experience** |  |  |  |  |  |  |  |  |  |
| **Non-User** | .175 (.040) | -7.56 | .000 | .112 to .275 |  | -.486 (.098) | -4.95 | .000 | -.679 to -.293 |
| **Private Service User** | .219 (.053) | -6.23 | .000 | .136 to .353 |  | .092 (.117) | 0.79 | .429 | -.137 to .321 |
| **Health Status** |  |  |  |  |  |  |  |  |  |
| **Medication** | 1.773 (.365) | 2.78 | .005 | 1.185 to 2.654 |  | .039 (.098) | .40 | .687 | -.153 to .232 |
| **High Blood Pressure, Diabetes** | 1.221 (.290) | .84 | .400 | .767 to 1.943 |  | .088 (.113) | .78 | .436 | -.134 to .310 |
