# Supplementary material for: Willingness to Use and Pay for Digital Health Care Services According to 4 Scenarios: Results from a National Survey
Source: JMIR Mhealth Uhealth. 2023 Mar 29;11:e40834. doi: 10.2196/40834 (PMC10131682; doi:10.2196/40834)
Supplement: Multimedia Appendix 3 [file mhealth_v11i1e40834_app3.docx]

**Multimedia Appendix 3**

WTU and WTP on Scenario C (Chronic disease situation & Expert management)

|  | **Willing to Use** | | | |  | **Willing to Pay** | | | |
| --- | --- | --- | --- | --- | --- | --- | --- | --- | --- |
|  | **OR(SE)** | **z** | **P** | **95% CI** |  | **Coef.(SE)** | **t** | **P** | **95% CI** |
| **Demographics** |  |  |  |  |  |  |  |  |  |
| **Age** | .982 (.008) | -2.13 | .033 | .966 to .999 |  | -.004 (.004) | -1.12 | .261 | -.012 to .003 |
| **Gender** | .705 (.119) | -2.07 | .038 | .507 to .981 |  | .250 (.080) | 3.12 | .002 | .093 to .408 |
| **Income** | 1.091 (.061) | 1.56 | .118 | .978 to 1.218 |  | .030 (.026) | 1.15 | .251 | -.021 to .080 |
| **Residence** | .911 (.162) | -.52 | .601 | .643 to 1.292 |  | -.076 (.082) | -.93 | .354 | -.236 to .085 |
| **Service Experience** |  |  |  |  |  |  |  |  |  |
| **Non-User** | .413 (.094) | -3.88 | .000 | .264 to .646 |  | -.336 (.096) | -3.51 | .000 | -.524 to -.148 |
| **Private Service User** | .401 (.098) | -3.74 | .000 | .249 to .647 | s | .264 (.114) | 2.32 | .021 | .040 to .488 |
| **Health Status** |  |  |  |  |  |  |  |  |  |
| **Medication** | 1.310 (.296) | 1.19 | .233 | .841 to 2.040 |  | .100 (.096) | 1.04 | .299 | -.089 to .288 |
| **High Blood Pressure, Diabetes** | 1.751 (.487) | 2.02 | .044 | 1.015 to 3.019 |  | .047 (.108) | .043 | .667 | -.166 to .259 |
