# Supplementary material for: Willingness to Use and Pay for Digital Health Care Services According to 4 Scenarios: Results from a National Survey
Source: JMIR Mhealth Uhealth. 2023 Mar 29;11:e40834. doi: 10.2196/40834 (PMC10131682; doi:10.2196/40834)
Supplement: Multimedia Appendix 4 [file mhealth_v11i1e40834_app4.docx]

**Multimedia Appendix 4**

WTU and WTP on Scenario D (Chronic disease situation & Medical management)

|  | **Willing to Use** | | | |  | **Willing to Pay** | | | |
| --- | --- | --- | --- | --- | --- | --- | --- | --- | --- |
|  | **OR(SE)** | **z** | **P** | **95% CI** |  | **Coef.(SE)** | **t** | **P** | **95% CI** |
| **Demographics** |  | z |  |  |  |  |  |  |  |
| **Age** | .967 (.009) | -3.55 | .000 | .950 to .985 |  | -.010 (.004) | -2.70 | .007 | -.018 to -.003 |
| **Gender** | .569 (.106) | -3.03 | .002 | .395 to .820 |  | .177 (.779) | 2.27 | .024 | .024 to .330 |
| **Income** | 1.040 (.065) | .063 | .529 | .921 to 1.175 |  | .334 (.025) | 1.34 | .181 | -.016 to .082 |
| **Residence** | .705 (.143) | -1.73 | .084 | .474 to 1.048 |  | -.125(.078) | -1.59 | .111 | -.279 to .029 |
| **Service Experience** |  |  |  |  |  |  |  |  |  |
| **Non-User** | .480 (.120) | -2.94 | .003 | .294 to .783 |  | -.286 (.092) | -3.10 | .002 | -.467 to -.105 |
| **Private Service User** | .345 (.089) | -4.11 | .000 | .208 to .573 |  | .193 (.111) | 1.73 | .084 | -.026 to .411 |
| **Health Status** |  |  |  |  |  |  |  |  |  |
| **Medication** | 1.500 (.390) | 1.56 | .119 | .901 to 2.498 |  | .037 (.092) | .40 | .690 | -.144 to .217 |
| **High Blood Pressure, Diabetes** | 1.894 (.596) | 2.03 | .042 | 1.022 to 3.510 |  | .103 (.105) | .98 | .326 | -.103 to .310 |
